# Applicability of three-dimensional imaging techniques in fetal medicine*

**DOI:** 10.1590/0100-3984.2015.0100

**Published:** 2016

**Authors:** Heron Werner Júnior, Jorge Lopes dos Santos, Simone Belmonte, Gerson Ribeiro, Pedro Daltro, Emerson Leandro Gasparetto, Edson Marchiori

**Affiliations:** 1 PhD, MD, Radiologist at the Alta Excelência Diagnóstica and at the Clínica de Diagnóstico Por Imagem (CDPI), Rio de Janeiro, RJ, Brazil.; 2 PhD, Technologist at the Instituto Nacional de Tecnologia, Rio de Janeiro, RJ, Designer at the Center for Three-Dimensional Experimentation of the Pontifícia Universidade Católica do Rio de Janeiro (PUC-Rio), Rio de Janeiro, RJ, Brazil.; 3 Biologist at the Center for Three-Dimensional Experimentation of the Pontifícia Universidade Católica do Rio de Janeiro (PUC-Rio), Rio de Janeiro, RJ, Brazil.; 4 Designer at the Center for Three-Dimensional Experimentation of the Pontifícia Universidade Católica do Rio de Janeiro (PUC-Rio), Rio de Janeiro, RJ, Brazil.; 5 PhD, MD, Radiologist at the Clínica de Diagnóstico Por Imagem (CDPI), Rio de Janeiro, RJ, Brazil.; 6 PhD, Full Professor of Radiology at the Universidade Federal do Rio de Janeiro (UFRJ), Rio de Janeiro, RJ, Brazil.

**Keywords:** Fetus, Fetal medicine, Three-dimensional technique, Ultrasound, Magnetic resonance imaging, Computed tomography

## Abstract

**Objective:**

To generate physical models of fetuses from images obtained with three-dimensional ultrasound (3D-US), magnetic resonance imaging (MRI), and, occasionally, computed tomography (CT), in order to guide additive manufacturing technology.

**Materials and Methods:**

We used 3D-US images of 31 pregnant women, including 5 who were carrying twins. If abnormalities were detected by 3D-US, both MRI and in some cases CT scans were then immediately performed. The images were then exported to a workstation in DICOM format. A single observer performed slice-by-slice manual segmentation using a digital high resolution screen. Virtual 3D models were obtained from software that converts medical images into numerical models. Those models were then generated in physical form through the use of additive manufacturing techniques.

**Results:**

Physical models based upon 3D-US, MRI, and CT images were successfully generated. The postnatal appearance of either the aborted fetus or the neonate closely resembled the physical models, particularly in cases of malformations.

**Conclusion:**

The combined use of 3D-US, MRI, and CT could help improve our understanding of fetal anatomy. These three screening modalities can be used for educational purposes and as tools to enable parents to visualize their unborn baby. The images can be segmented and then applied, separately or jointly, in order to construct virtual and physical 3D models.

## Abstract (pt)

**Objetivo:**

Gerar modelos físicos de fetos utilizando imagens obtidas por ultrassonografia tridimensional (US3D), ressonância magnética (RM) e, em alguns casos, tomografia computadorizada (TC), para orientar a técnica de adição de camadas.

**Materiais e Métodos:**

Foram usadas imagens obtidas de 31 gestantes, incluindo 5 casos de gestação gemelar. Os exames foram realizados usando US3D, RM e em alguns casos TC, e os arquivos foram exportados para uma estação de trabalho em formato DICOM. Um único observador realizou o processo de segmentação manual usando tela de alta resolução. Um *software* que converte imagens médicas em modelos numéricos foi utilizado para construir modelos virtuais 3D, que foram fisicamente materializados.

**Resultados:**

Os modelos virtuais e físicos baseados na US3D, RM e TC realizados separadamente ou em conjunto foram concluídos com sucesso. A aparência pós-natal do feto abortado ou do recém-nascido se assemelhou muito com os modelos físicos, particularmente nos casos de malformações.

**Conclusão:**

O uso da US3D, RM e TC pode ajudar para melhor compreensão das características físicas do feto. Essas técnicas podem ser usadas com fins didáticos para auxiliar na abordagem multidisciplinar e na melhor compreensão dos pais. As imagens podem ser segmentadas e aplicadas separadamente ou combinadas para construir modelos virtuais 3D e físicos.

## INTRODUCTION

A growing number of technological advancements in obtaining and viewing images through noninvasive techniques have brought major breakthroughs in medicine, especially in the diagnosis of fetal anomalies^(1,2)^. In general, two types of examinations are used in order to obtain images of the uterine cavity during pregnancy^(1,3)^: ultrasound and magnetic resonance imaging (MRI). Computed tomography (CT) also provides detailed images of the fetus, especially of its skeleton, from the 30th week of pregnancy, although its utility is restricted because it involves the use of ionizing radiation^(4)^.

Three-dimensional (3D) virtual modeling has gained great momentum in recent years, due to the high performance of software applied in the fields of engineering, architecture, and design. It has been taking an increasingly userfriendly form, facilitating the visualization of 3D images^(5,7)^.

The objective of this study was to develop virtual 3D models of fetuses during pregnancy from images obtained by ultrasound, MRI, and CT, alone or in combination.

## MATERIALS AND METHODS

This study evaluated 31 pregnant women between January 2008 and December 2014. The study was approved by the Research Ethics Committee of the Instituto Fernandes Figueira (IFF/Fiocruz). All the patients involved underwent 3D ultrasound (3D-US), alone or in combination with MRI, with no more than one day between the examinations in the latter case (Table 1). In all cases of suspected fetal malformation based on a previous ultrasound, the combination of MRI and 3D-US was used. All 3D reconstructions for prototyping were performed at National Institute of Technology and at the Center for Three-Dimensional Experimentation of the Pontifícia Universidade Católica do Rio de Janeiro, both of which are also located in the city of Rio de Janeiro, Brazil.

**Table 1:** Summary of the five cases in which CT was used.

Case	Gestational age (weeks)	Diagnosis	Method	Technique
1	34	Hypoplasia of the left femur	3D-US / CT	SLA
2	34	Hypoplasia of the femur and left tibia, together with left fibular agenesis	3D-US / CT	SLA
3	34	Achondrogenesis	3D-US / MRI / CT	SLA, ZCorp
4	34	Thoraco-omphalopagus	3D-US / MRI / CT	ZCorp
5	35	Amputation of the legs, ectrodactyly of the right hand, and syndactyly of the left hand	3D-US / MRI / CT	ZCorp

SLA, stereolithography, liquid-based system; ZCorp, powder-based system.

The following inclusion criteria were applied: singleton or multiple gestation with gestational age established by an ultrasound performed up to the 16th week of pregnancy or based on the date of the last menstrual period in women with a history of regular cycles; and fetuses with suspicion of abnormality or malformation, as identified by ultrasound.

All of the pregnant women evaluated were at least 18 years of age and were examined between the 22nd and 37th weeks of gestation, some being scheduled for a second examination, as necessary. Ultrasound and MRI examinations were performed and monitored by two professionals: a specialist in gynecology, obstetrics, and fetal medicine; and a specialist in radiology.

The equipment used in the examinations were the Voluson 730 and Voluson E8 ultrasound systems (GE Medical Systems/Kretztechnik GmbH, Zipf, Austria), with a 4-8 MHz transvaginal/transabdominal transducer. The MRI scans were obtained on one of two types of 1.5 T scanners (Magnetom Avanto and Aera; Siemens Healthcare, Erlangen, Germany). Patients were placed in the supine or left lateral decubitus position, whichever made them more comfortable, and were introduced into the scanner feet first, in order to reduce the feeling of claustrophobia. A surface coil was positioned over the abdomen of the pregnant woman, and the following protocol was applied: T2-weighted HASTE sequences-repetition time/echo time (TR/TE), 140/140 ms; field of view (FOV), 300-200 mm; gap, 0; matrix, 256 × 256 mm; 4-mm slices; acquisition time, 18 seconds; and 40 slices in the axial, coronal, and sagittal planes of the fetus)-and 3D volumetric (TrueFISP) sequences-TR/TE, 3.02/1.34 ms; FOV, 340 mm; matrix, 256 × 90-256 mm; slices of 1.0-1.6 mm; acquisition time, 26 seconds; and 96-196 slices, preferably in the sagittal plane of the fetus. Each examination was completed in 40 minutes or less^(1,3)^.

In five cases of fetal skeletal malformations, we used files from CT scans obtained after the 30th week of pregnancy (Table 1). Those scans were obtained with a 64-channel multislice tomograph (Brilliance; Philips, Solingen, Germany), with the following parameters: 40 mAs, 120 kV, 64 slices/rotation, a pitch of 0.75, and slices of 0.75 mm. That corresponds to a mean radiation dose of 3.12 mGy, dose-length product of 160.3 mGy.cm, and effective dose of 2.40 mSv^(4,5)^.

For the construction of the physical model from 3D-US, MRI, and CT data, the first step was to create the 3D virtual model of the fetus. All images generated by 3D-US, MRI, and CT were exported to a workstation in the DICOM format. The segmentation was then achieved by a technician with experience in 3D modeling, under the supervision of the physician in charge. The fetuses were reconstructed from thin slices that, collectively, generated a 3D surface, soft tissue information being obtained by 3D-US, MRI, or both, CT providing information only related to the skeletal structure. Segmentation by 3D-US was performed in all cases (Tables 1, 2, and 3). For the segmentation of medical images, we used the software Mimics, version 12 (Materialize, Leuven, Belgium), generating the final virtual model in the "wavefront object" and "standard triangular language" formats, the latter intended for 3D printing.

**Table 2:** Summary of four cases of multiple pregnancy with the use of 3D-US and MRI.

Case	Gestational age (weeks)	Diagnosis	Method	Technique
6	31	Twin pregnancy (one fetus with agenesis of the corpus callosum)	3D-US / MRI	SLA, ZCorp
7	28	Twin pregnancy (one fetus with ventricular dilatation)	3D-US / MRI	SLA, ZCorp
8	27	Diencephalic syndrome	3D-US / MRI	ZCorp
9	27	Triplet pregnancy	3D-US / MRI	ZCorp

SLA, stereolithography, liquid-based system; ZCorp, powder-based system.

**Table 3:** Summary of 21 cases of singleton gestation involving 3D-US associated with MRI.

Case(s)	Gestational age (weeks)	Diagnosis	Method	Tecnnique
10	26	Chiari II	3D-US / MRI	SLA, ZCorp
11	29	Agenesis of the corpus callosum	3D-US / MRI	SLA, ZCorp, FDM
12, 13	28, 32	Cleft lip	3D-US / MRI	ZCorp
14	31	Diaphragmatic hernia	3D-US / MRI	SLA, ZCorp
15, 16	26, 25	Alobar holoprosencephaly	3D-US / MRI	SLA, ZCorp
17	34	Hydrocephalus	3D-US / MRI	SLA, ZCorp
18	26	Trisomy 21	3D-US / MRI	ZCorp
19	30	Sacrococcygeal teratoma type III	3D-US / MRI	SLA, ZCorp
20	26	Beckwith–Wiedemann syndrome	3D-US / MRI	ZCorp
21	22	Encephalocele	3D-US / MRI	ZCorp
22, 23	27, 28	Lymphangioma	3D-US / MRI	ZCorp
24	37	Cervical teratoma	3D-US / MRI	ZCorp, Objet Connex
25	32	Apert syndrome	3D-US / MRI	ZCorp
26	26	Thanatophoric dysplasia	3D-US / MRI	ZCorp
27	29	Translocation 7;15	3D-US / MRI	ZCorp
28	28	Retrognathism	3D-US / MRI	ZCorp
29	30	Left radial agenesis and omphalocele	3D-US / MRI	ZCorp
30	32	Esophageal atresia	3D-US / MRI	ZCorp, Objet Connex
31	32	Epignathus	3D-US / MRI	ZCorp

SLA, stereolithography, liquid-based system; ZCorp, powder-based system; FDM, fusion deposition modeling.

The process of reconstruction of fetuses in physical models from ultrasound, MRI, and CT images g;nerated a patent (serial number PI08090521).

## RESULTS

**Figure 1:**
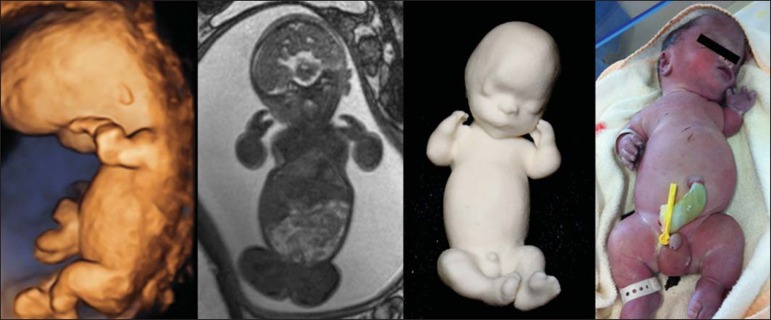
*Case 26*. Fetus with thanatophoric dysplasia. Ultrasound, MRI, physical model, and stillborn fetus.

**Figure 2:**
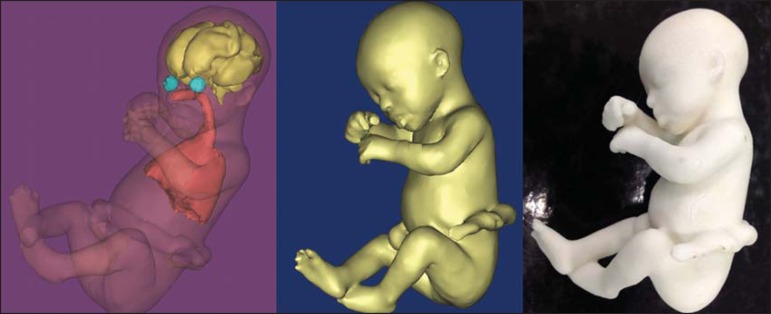
*Case 18*. Fetus with trisomy 21. Whole-body virtual and physical model obtained by MRI.

**Figure 3:**
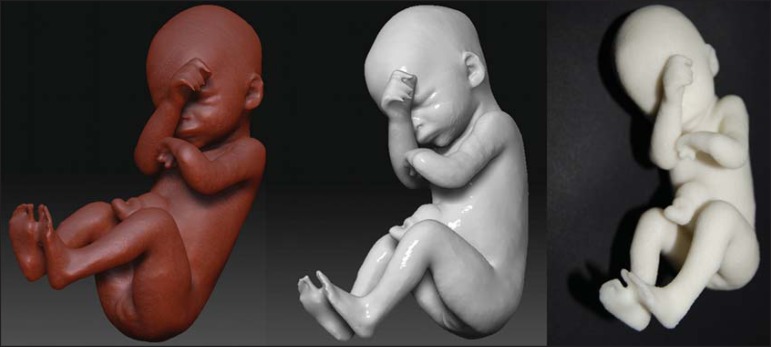
*Case 29*. Fetus with left radial agenesis and omphalocele. Whole-body virtual and physical model obtained by MRI.

The physical models generated were considered satisfactory in all cases (Figures 1, 2, 3, and 4). The average printing time and cost for each process are summarized in Table 4. CT provided high-resolution images of the fetal skeleton. MRI images showed high contrast between the organs and external surface. The physical models obtained by 3D-US provided excellent data for the impressions of the face, ears, hands and feet.

**Figure 4:**
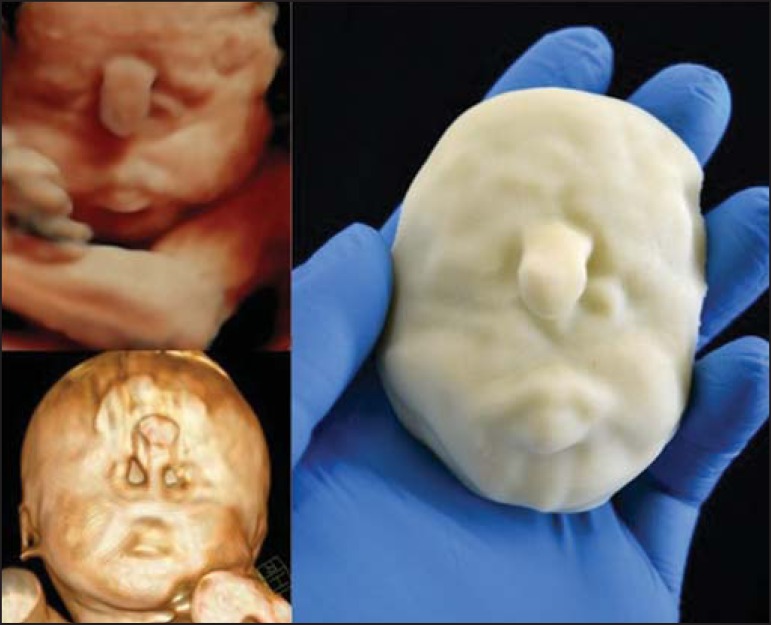
*Case 16*. A: Fetus at the 25th week of gestation, showing alobar holoprosencephaly and proboscis. Face obtained by 3D-US/3D MRI, and physical model obtained from the ultrasound data.

**Figure 4B:**
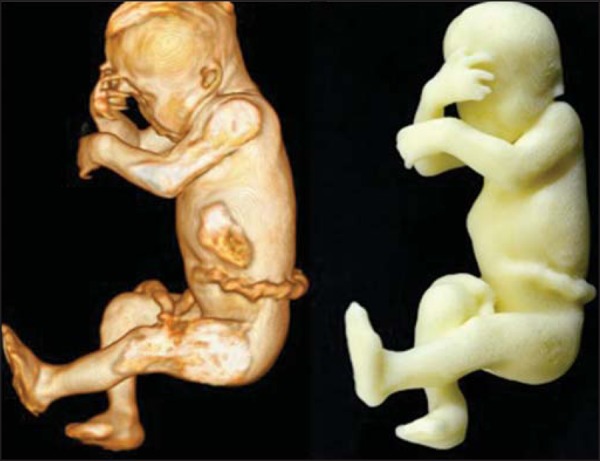
Whole-body 3D reconstruction from the MRI and physical model.

**Figure 4C:**
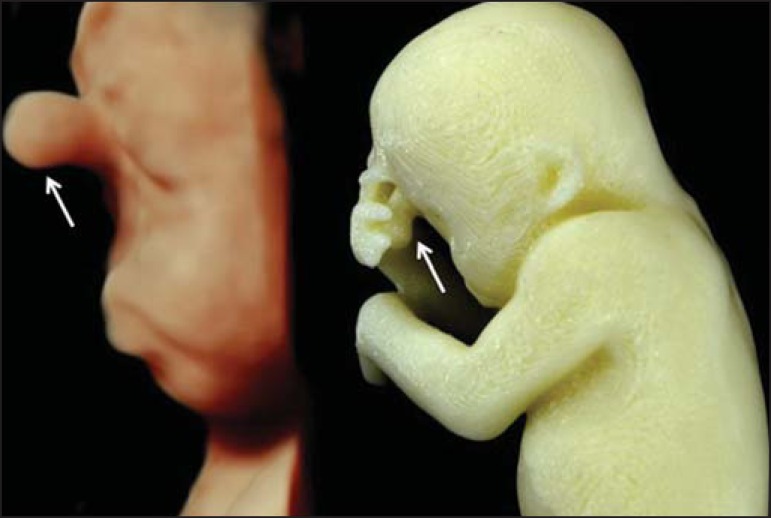
Fetal profile obtained by 3D-US and physical model. Notice the proboscis (arrow).

**Figure 4D:**
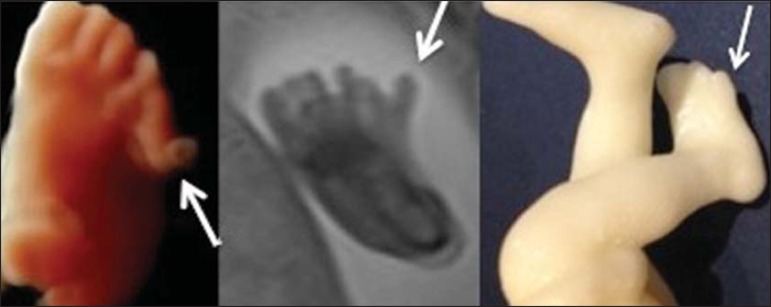
Polydactyly of the foot identified by 3D-US, by MRI, and in the physical model.

**Table 4:** Estimated time and cost of manufacturing in all 31 of the cases evaluated.

Case(s)	Estimated time (hours)	Estimated cost (US$)
1, 2, 8, 9, 10, 11, 16, 23, 24,		
25, 29, 30, 31	22–26	1300–2500
12, 19, 20, 21, 22	2–4	80–120
14, 26	5–7	200–400
13, 28	1–2	30–80
6, 15, 17, 18	4–5	150–250
3	11	800
4	28	1900
5, 7, 27	7–8	280–500

A combination of methods for the construction of physical models was successfully devised. In the case of a 25-week fetus with alobar holoprosencephaly and proboscis (case 16, Table 3), which had been assessed by 3D-US and MRI on the same day, the body was modeled based on the MRI file, whereas the face and the extremities were modeled based on the 3D-US file. In that case, the 3D-US was instrumental in the evaluation of the extremities and the face (Figure 4).

## DISCUSSION

Additive manufacturing technology allows the conversion of a virtual 3D model to a physical model, with precise dimensions, in a process that is fast and easy. The process transfers a 3D data file, obtained by superimposing individually segmented layers, to an additive manufacturing device, or 3D printer, which constructs physical models by superimposing thin layers of raw materials^(6,9)^.

The main finding of the present study was that it is possible to create virtual and physical 3D models from 3D-US or MRI data, although the combination of the two was required in cases that were more complex and could not be reliably diagnosed by ultrasound. The inclusion of CT data in five cases was justified because the initial phase of the development of this technique of 3D reconstruction of a fetus in a physical model involved CT data only. Only thereafter were the other versions of the technique mastered, first the MRI-based version and then the ultrasound-based version^(10,11)^.

Werner et al.^(10)^ introduced the use of physical models in fetal disease research, an area in which studies involving digital (3D) modeling are scarce^(11,14)^. The results suggest a new possibility in the interaction between the parents and the fetus during prenatal monitoring, physically recreating the interior of the uterus during pregnancy, demonstrating the actual size of the fetus, as well as its anatomy.

One of the main concerns of this study was to obtain high-quality images that can be manipulated with 3D software, without a loss of accuracy. Fetal movements during the acquisition of images constituted one of the main difficulties, especially in the MRI evaluation. This problem is minimized in ultrasound, because the image is acquired in real time and can be frozen during movement. However, in some cases, the lower resolution of contrast ultrasound created difficulties due to the limits of the gray scale. The quality of the process is directly associated with the accuracy of the mathematical data that will be used in order to generate the physical model. The images are acquired in slices, which are superimposed for the construction of the model.

The physical models have an impact on the planning of medical interventions^(15,16)^. The can also be used in fetal medicine for educational purposes^(11,14,17,18)^. The act of combining images obtained by different methods (ultrasound and MRI) can result in better understanding, on the part of the parents and of a multidisciplinary medical team, in evaluating certain types of diseases^(11,14)^.

Previous studies have employed ultrasound and 3D models. Blaas et al.^(19)^ calculated the volumes of embryos and fetuses in the first trimester of pregnancy, transforming the embryo/fetus area into a virtual model. In another study, conducted by Nelson et al.^(20)^, 3D-US data were converted into a set of polygons representing a surface that could be transferred to various types of rapid prototyping equipment, in order to create a solid 3D object. That was considered the first attempt to transform 3D-US data into physical models. In the present study, we attempted to demonstrate the advantages of 3D visualization over traditional images. In 3D visualization, the area of interest can be clearly evaluated and manipulated by the observer, who can thus appreciate the physical characteristics of the object and their spatial relationships. Therefore, the additive manufacturing device, or 3D printer, came to function as a 3D display device, representing a powerful tool to facilitate the visualization of various anatomical structures. The models generated represented an important means of communicating, in a tangible way, with pregnant women, providing them with information that is more easily understood.

Based on those experiments, the study of 3D fetal modeling started using CT files related to fetuses with a gestational age over 30 weeks in order to build physical models of the fetal skeleton^(7,11)^. The result was a series of connecting structures of bones in a virtual 3D environment^(6)^. To maintain the integrity of the entire virtual skeleton, with preservation of its shape and spatial coordinates, modeling was performed with software (Autodesk Maya; Alias/Wavefront, Santa Barbara, CA, USA) that allowed a physical model to be produced without losing the accurate positioning of its different parts. The next challenge was the delimitation of the entire outer surface of the fetal body based on the slices obtained by CT. This interactive visual process detected the limits of fetal body parts using a digital stylus, which interacts directly with the computer screen. The resulting layers of the entire fetal surface were superimposed, generating a 3D volumetric model.

Based on the results obtained with the CT files, studies using files obtained from fetal MRI scans were initiated. Although manual segmentation was used, the biggest problem with the MRI-based technique was the thickness of the slices and the smaller number of slices. The main difference between the data obtained by CT and those obtained by MRI was the quality of the contrast between the organs. The grayscale contrast between the organs is greater in MRI. This greater sharpness allowed easy visual separation of the relevant areas on a graphics processing screen with a variablepressure stylus. On CT scans, only the skeleton was easily identifiable. However, despite the better contrast obtained with MRI, there was at first a limitation on the number of slices obtained (approximately 30-40), making the accuracy of the final result questionable. However, with the use of the TrueFISP sequence, which offered a larger number of slices (> 90), the fetal outline obtained became clearer. The TrueFISP technique allowed the slice thickness to be reduced from 4 mm to approximately 1 mm. In the case of MRI, it was easier to obtain images of better quality at later gestational ages, when there is less interference from motion artifacts.

The biggest challenge was in the construction of models using ultrasound^(7)^. The ultrasound modality allows a faster scan of the fetus, the image being automatically transformed into a virtual 3D image on the screen^(21,23)^. Up to the 18th week of gestation, ultrasound allows complete viewing of the fetal body. Thereafter, the fetal body parts are visualized as separate blocks to be joined. The tomographic ultrasound imaging function (ultrasound images in CT form) of the 4D View GE software was used in order to render the 3D-US images, obtaining results similar to those obtained by MRI. The images were exported to the Mimics software for 3D image reconstruction, maintaining the accuracy and reliability. Thus, the MRI protocol was adopted for the processing of ultrasound images.

The mastery of the ultrasound reconstruction technique opened up the possibility of combining the MRI and ultrasound files when they were acquired on the same day^(11)^. In that way, all 3D files obtained by ultrasound, MRI, and, in some cases, CT could be combined. As exemplified in case 13 of the present study, it became possible to combine 3D-US images of the face, hands, or feet with fetal body images obtained by MRI, and the necessary biometric proportions can be maintained by means of various measures for both techniques.

As for the cost of production of the physical models, the four manufacturing techniques adopted in this case study differed in relation to the construction and the materials used, which are the main items to be considered in the calculation of costs^(11)^. The most widely used technique involved printers using ZCorp (plaster-based composite) powder. The physical models resulting from that process are the least expensive, especially when compared with those resulting from processes such as stereolithography and selective laser sintering, which use a laser beam for hardening a photosensitive resin layer or sintering polyamide powder^(6,7)^.

## CONCLUSION

The segmentation and reconstruction techniques developed for fetal modeling can be applied to the construction of virtual and physical models obtained from ultrasound, MRI, and CT images, individually or in combination.

On the basis of the results of this study, we believe that the physical models will, in the near future, facilitate the tactile and interactive study of complex abnormalities in various disciplines. These techniques may also be useful for prospective parents, to recreate a 3D model with the physical characteristics of the fetus, allowing a more direct emotional connection with the unborn child.
